# 

**Published:** 1873-07

**Authors:** 


### CONTENTS.

ORIGINAL COMMUNICATIONS.

Pneumonia Acute—Its Treatment, etc. By A. S. Payne, M.D., . . . 385
Clinical Report of Stray Cases of Constitutional Syphilis. By T. Curtis Smith, M.D., . . . . . . . . . . . . . . . 403
Medical Observations. By Fred. Horner, Jr., M.D., . . . . . 412
Is this Woman Pregnant? By Robert Battey, M.D., . . . . . . 417
Epileptic Mania Following the Suspension of Bromide Potash. By G. D. Hodge, M.D., . . . . . . . . . . . . . 420

EXTRACTS AND GLEANINGS.

Ferric Alum in Hemorrhagica; Calabar Bean—421. Propylamin Revived as a Cure for Rheumatism; Chloral Hydrate in Puerperal Convulsions; Formula for Podophyllin—422. Acne Rosacea; Vehicle for the Internal Administration of Chloroform—423. On the Use of Phosphorus in Certain Diseases of the Skin; How to Deprive Iodine of its Stain; New Anodyne Colloid—424. Toothache; Styptic Collodion; Case of Beriberi—425. Gelseminum in Gynæcology; Chronic Conjunctivitis in Children; Treatment of Hooping-Cough—426. Oulmont on Treatment of Mercurial Tremors and Paralysis Agitans by Hyoscyamine; Pulv. Rhei Co. Granulata; Cornea Affections—427. Little on the Use of Digitalis in the Failing Heart and Delirium of Acute Diseases; Local Employment of Chlorate of Potash in Cancerous Sores; Boils, their Cause and Cure—428. Treatment of Warts; Liquor Picis Alkalinus; Cerebro-Spinal Meningitis—429. New Operation for the Radical Cure of Hernia; Night Sweats of Phthisis; Eczema—430. Detection of Indican in the Urine; Siredey and Cordier on the Treatment of Anasarca, Ascites, and Obstinate Pleuritic Effusions by Milk; Nature and Treatment of Cholera—431. The Employment of Pessaries; Insard on the Treatment of Constipation by Arsenic; Treatment of Small Pox by Use of Tinct. Ferri Chloridi—432. Carbolic Acid in Skin Diseases; Neuralgia; Treatment of Consumption at the Brompton Hospital; Chronic Eczema of the Extremities—433. Orchitis Treated with Nitrate of Silver; Rheumatism, Cause and Treatment; Digitalis an Anaphrodisiac; Oxide of Zinc for Night Sweats—434. Use of Actea in Lumbago and Rheumatism; Anodyne Colloid; Potassium Chlorate in Catarrh; Hemoptysis, Gallic Acid—435. Phosphorus Mixture; Treatment of Chronic Disease of the Bladder by the Injection of Healthy Urine; Salicine in Obstinate Diarrhœa; Belladonna in Intestinal Invagination and Hernia—436. Convulsions in a New-Born Child from Alcoholism of the Nurse; Ethiops Mineral in Cholera; The Pulse in Childhood; Chloroform in Lead Colic—437. Mercury in the Treatment of Bronchitic Asthma; Treatment of Paralysis Agitans; Oxide of Zinc Ointment; Vehicle for Chloral Hydrate—438. Acorn Coffee in Diarrhœa; Therapeutical Additions; Ergot in Phthisical Hemoptysis; Colorless Tincture of Iodine—439. Treatment of Toitre; Triplex Pill of Dr. John Francis; Elongation of the Uvula—440. Treatment of Typhoid Fever; Herpes Preputialis; Sick Headache; Cold Baths in Rheumatic Fever; A Local Anæsthetic—441. Post-Partem Hemorrhage; Determination of the Life or Death of the Fœtus; Opium Antidotes; Chloral after Chloroform; Local Styptic—442. Toothache Mixture; Hydrate of Chloral in Labor; Gleet and Chronic Irritation of the Urethra; Fistula in Ano—443.

EDITORIAL AND MISCELLANEOUS.

Advertising Department; Books, . . . . . . . . . 444
Medical Colleges, . . . . . . . . . . . . . 445
Our Original Department; Cincho-Quinine, . . . . . . . . 446
Ponce de Leon Spring; Dr. P. T. Y., Beautiful Chromos, . . . . 447

